# Combining blood meal analysis and parasite detection yields a more comprehensive understanding of insect host feeding patterns

**DOI:** 10.1186/s13071-025-06931-8

**Published:** 2025-07-20

**Authors:** Anna Kapustová, Magdaléna Kulich Fialová, Milena Svobodová, Jana Brzoňová

**Affiliations:** Charles University, Prague, Czechia

**Keywords:** Mosquito, Biting midge, Blood meal, Host feeding patterns, Avian trypanosomes, Haemosporidians, *T. theileri*, Method comparison

## Abstract

**Background:**

Traditionally, blood meal analysis has been the primary method used to assess feeding patterns of insects. In contrast, parasite detection is commonly applied to monitor parasite circulation and prevalence in vectors, but rarely to study host feeding patterns. Our study aimed to test whether broad-target screening for haemosporidian and trypanosome parasites could complement blood barcoding by revealing additional host associations. We hypothesised that combining both methods would provide a more comprehensive understanding of vector feeding behaviour than either method alone. In addition to evaluating the two methods, we also analysed the vector species composition and their abundance, providing important faunistic and prevalence data that contribute to the broader understanding of local vector–parasite dynamics.

**Methods:**

Mosquitoes and biting midges were trapped over a 5-year period at three localities in Czechia. Blood-fed individuals underwent blood meal barcoding analysis. In parallel, parasite detection was conducted using nested polymerase chain reaction (PCR) and gut dissection techniques.

**Results:**

A total of 10,152 mosquitoes were collected, with *Culex pipiens* (66%) and *Aedes vexans* (18%) being the predominant species. In addition, 1701 biting midges, primarily *Culicoides pictipennis* (61%) and *C. festivipennis* (12%), were captured. Among the collected samples, 281 mosquitoes (3%) and 52 biting midges (3%) were blood-fed. Parasites were detected in 468 mosquito pools (5%, 341 trypanosomes, 127 haemosporidians) and 21 midge pools (1%, 8 trypanosomes, 13 haemosporidians). Blood meal barcoding of engorged *Aedes*, *Anopheles*, *Culiseta,* and *Mansonia* samples revealed only mammalian hosts; however, parasite detection indicated previous feeding on birds. *Culex* displayed stronger ornithophily according to parasite detection, although blood meal analysis showed a more opportunistic behaviour, with the detection of avian, mammalian and even amphibian blood. Avian parasites were detected in five *Culicoides* species (*Culicoides alazanicus*, *C. festivipennis*, *C. kibunensis*, *C. nubeculosus* and *C. pictipennis*) while human blood was detected only in *C. pictipennis*. Overall, four *Haemoproteus* lineages and 15 *Plasmodium* lineages were identified, 11 of which were new records for Czechia and 4 were newly described.

**Conclusions:**

Integrating blood meal analysis with parasite detection provides a more comprehensive understanding of insect feeding patterns and vector–host dynamics. Blood meal analysis remains the gold standard for identifying recent host interactions, offering direct and often species-level evidence of feeding events. In addition, parasite detection extends the window of detectability beyond the digestion of host blood and can reveal additional or otherwise-overlooked host associations. Together, these complementary approaches increase the likelihood of detecting interactions with a broader range of hosts, including humans, who might be missed by parasite screening alone.

**Graphical Abstract:**

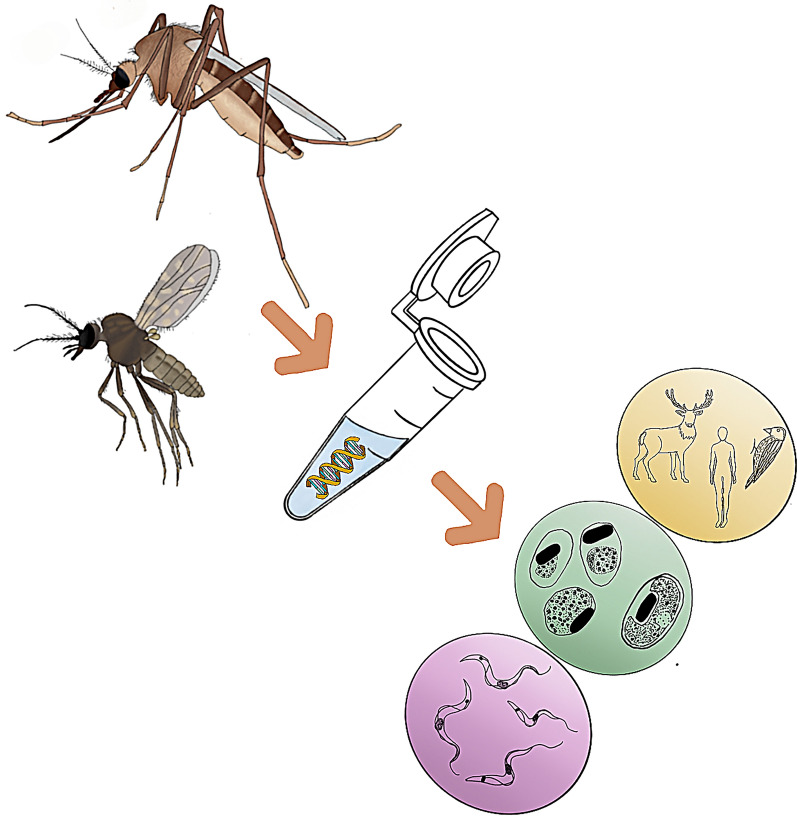

## Background

Many species of blood-feeding Diptera are essential vectors of pathogens that affect both humans and animals. These insects can transmit viruses, bacteria, protozoa and helminths, all of which are capable of causing serious diseases. Studying the behaviour and feeding patterns of vectors is an important step in understanding the exposure of vertebrate hosts to vector-borne diseases.

Several methods can be used to reveal the feeding patterns of blood-feeding insects, such as host choice tests using experimental animals [1–3] or the analysis of blood from naturally engorged insects caught in traps [2, 4–6]; however, each method has certain limitations. Choice experiments are limited by the availability of experimental animals and exclude a wide range of potential hosts found in natural environments. Blood analysis is constrained by the typically low numbers of fed females caught in traps and by the rapid degradation of ingested blood, which makes it difficult to determine the origin of the blood meal [7, 8].

Another indirect approach to investigate the feeding patterns of vectors is through the detection of parasites. Blood-sucking insect species differ in their feeding behaviour, with some preferring mammals, and other birds or cold-blooded hosts. These specific feeding patterns can limit the spread of parasites to certain host groups. However, some vector species have broad feeding habits, linking a wide range of hosts and facilitating pathogen transmission. For example, opportunistic feeding of *Culex* mosquitoes enables the transmission of West Nile virus from birds to mammals, including humans [2, 9, 10]. Parasite detection thus provides an alternative approach to studying the feeding patterns of haematophagous insects [11]. The main advantage of this method is that parasites remain detectable in vectors much longer than ingested blood [12], providing an extended window of opportunity for investigating host feeding patterns.

However, protocols used for blood-feeding insects often detect not only their specific parasites but also non-specific ones that are incapable of developing into transmissible stages for new hosts [13, 14]. Some of these non-specific parasites can persist even after the ingested blood has been fully digested [15, 16]. Nevertheless, such findings cannot be used to assess the vector’s transmission capacity. Despite that, the presence of non-specific parasites may still provide valuable information for studying vector–host relationships. Lineages of haemosporidia and trypanosomes found in vectors can help determine the feeding patterns of blood-feeding insects. Given the specificity of host–parasite relationships, it is sometimes possible not only to distinguish whether insects are mammalophilic or ornithophilic, but also to uncover more specific host–parasite interactions [11, 12].

In this study, we focused on the comparison and combination of two methods that reveal the host feeding patterns of blood-sucking insects, namely direct barcoding of ingested blood and parasite detection in potential vectors. We studied both mosquitoes and biting midges, and their haemosporidian parasites as well as trypanosomes. Besides comparing the two methods, we also investigated vector species composition and abundance, generating valuable faunistic and prevalence data that contribute to a better understanding of local vector–parasite interactions.

## Methods

### Collection sites

**Fig. 1:**
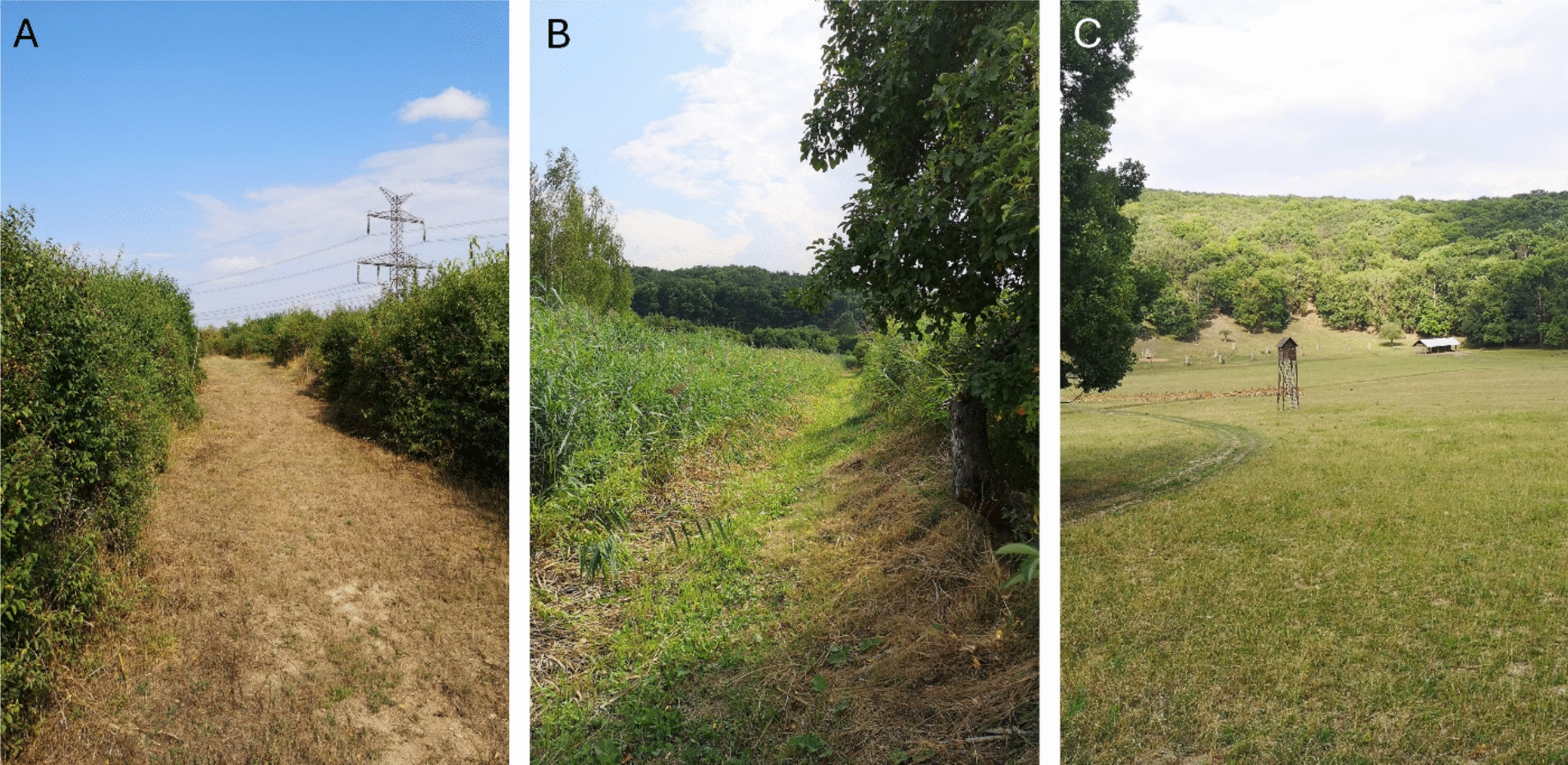
Localities used for blood-sucking insect collection: **A** Choteč, **B** Zeměchy, **C** Milovice forest

Insect collection took place at three different localities in Czechia: Choteč (49.9991° N, 14.2802° E), Zeměchy (50.2307°N, 14.2781°E) and Milovice forest (48.8213° N, 16.6932° E) (Fig. 1A–C). Choteč represents the driest site with the nearest water source about 1 km away, and it is characterised by successional shrubs such as blackthorn (*Prunus spinosa*), elderberry (*Sambucus* sp.), hawthorn (*Crataegus laevigata*) and plum (*Prunus cerasifera, P. domestica*). The Zeměchy site consists of reed beds (*Phragmites australis*) with a seasonal water stream (muddy/dried up during summer), and nearby shrubs such as elderberry (*Sambucus* sp.) and apple trees (*Malus* sp.) as well as surrounding forest. Milovice forest is a deer game reserve dominated by oak (*Quercus* sp.) and ash (*Fraxinus* sp.), but also contains multiple clearcuts and dry, extensively grazed meadows.

### Trapping methods, mosquito and biting midge identification

Haematophagous insects were trapped overnight at monthly intervals from May to August during the years 2017–2021. Each trapping event used six CDC light traps (JW Hock Company, Gainesville, FL, USA) without bulbs, baited with dry ice to release CO_2_ as an attractant. The traps were set up at fixed locations between 4:00 p.m. and 6:00 p.m. and were removed between 8:00 a.m. and 10:00 a.m. the following day. Collected insects were killed by freezing and stored at –20 °C until species determination. In addition, each July from 2017 to 2021, extra mosquito trapping was conducted in Milovice forest for gut dissection (see below).

Mosquitoes were identified under a stereomicroscope, on the basis of morphological characteristics [17]. Biting midges were determined according to their wing pattern and other morphological differences using an interactive key [18]. To find out the species composition of the *Culicoides obsoletus* complex, 20 randomly selected specimens were barcoded by analysing of the mitochondrial cytochrome *c* oxidase subunit I gene (*COI*) using LCO 1490 and HCO 2198 primers [19]. Biting midges were divided into parous or nulliparous, according to the red pigmentation of subcutaneous cells of the parous female’s abdomen, indicating a digested blood meal before capture [20]. Parasite-positive *Culicoides* that could not be morphologically identified were barcoded [19]. Mosquitoes without visible blood meals, parous females and dark biting midges (included owing to unclear parity status based on pigmentation) were pooled by species, trapping sites and trapping session into pools of up to ten individuals and screened molecularly for parasite presence. In contrast, insects with visible blood meals were processed individually for host identification and parasite screening (see below). Samples were stored at −20 °C prior to DNA extraction.

### Mosquito dissection

Mosquitoes were identified, killed and washed in 70% ethanol, followed by rinsing in sterile saline solution. The gut was dissected in a drop of sterile saline solution under a stereomicroscope and examined under a light microscope for the presence of trypanosomes and haemosporidian oocysts. Infected guts were preserved in ethanol for subsequent polymerase chain reaction (PCR) detection (see below).

### DNA extraction

DNA was extracted using the High Pure PCR Template Preparation Kit (Roche Diagnostic, Manheim, Germany) or E.Z.N.A.^®^ DNA/RNA Kit (OMEGA Bio-Tek, Norcross, GA, USA) according to the manufacturer’s instructions.

### Molecular detection of parasites and host blood sources

For *Trypanosoma* detection, the SSU rRNA gene was amplified using a specific nested PCR with S762 and S763 primers [21] for the first step and TR-F2 and TR-R2 [22] for the second one, as described by Brotánková [23].

For the detection of haemosporidian parasites (*Plasmodium*, *Haemoproteus* and *Leucocytozoon*), the cytochrome *b* gene fragment was amplified using nested PCR [24]. In cases where longer cytochrome *b* sequences were needed, the protocol by Perkins and Shall [25] was used.

To determine the host blood in blood-fed females, the primers 12S3F and 12S5R were used for the 12S mitochondrial rRNA gene amplification [26].

A negative control, a specimen without DNA was included every ten samples in each PCR run. Positive controls were included in each PCR run using *Leishmania major* DNA for *Trypanosoma* detection, *Plasmodium* sp. and *Haemoproteus* sp. DNA for haemosporidian detection, bird and mouse blood DNA for blood barcoding and *Cx. pipiens quinquefasciatus* DNA for insect barcoding.

Positive PCR products were purified using ExoSAP (Thermo Fisher Scientific, Inc., Waltham, MA, USA) and sequenced at the Laboratory OMICS—Genomics, Biocev. Geneious Sequence quality was assessed using Geneious Prime software, then the BLAST algorithm using NCBI or MalAvi nucleotide databases for sequence analysis/barcoding was used.

### Assessment of parasite prevalence (MIR)

The prevalence of parasites (in per cent) was calculated as a minimal infection rate (MIR) using the following formula:

$$MIR\left(\%\right)=\frac{number of positive pools}{number of examined insects}\bullet 100$$

This approach was chosen to efficiently detect parasite presence while making optimal use of available resources. Testing each insect individually was not feasible owing to time and cost constraints. MIR provides a standardised estimate of infection prevalence in pooled insect samples and is widely used for comparisons in vector surveillance studies.

## Results

### Abundance of insect species

#### Mosquitoes

A total of 10,152 mosquito females, representing 19 species, were trapped during the 2017–2021 seasons (Table 1). Overall, the most abundant mosquito species was *Culex pipiens* (*n* = 6747, 66%), followed by *Aedes vexans* (*n* = 1877, 18%) and *Mansonia richiardii* (*n* = 495, 5%). Mosquitoes were present at all localities, and *Culex* was the most abundant genus at every site.

**Table 1:** Mosquito species trapped during seasons 2017–2021

Species	No. trapped	No. of pools for analysis
*Ae. vexans*	1877	1060
*Ae.* sp.	276	83
*Ae. cantans/annulipes*	113	47
*Ae. punctor*	89	49
*Ae. caspius*	53	23
*Ae. cinereus*	76	15
*Ae. excrucians*	38	24
*Ae. rusticus*	7	6
*Ae. sticticus*	7	7
*Ae. flavescens*	4	1
*Ae. cataphylla*	2	2
*Ae. communis*	1	1
*An. plumbeus*	83	54
*An. maculipennis*	32	25
*An. claviger*	24	10
*Cx. pipiens*	6729	1796
*Cx. modestus*	14	11
*Cx.* sp.	4	4
*Cs. annulata*	215	147
*Cs. morsitans*	13	5
*Ms. richiardii*	495	178
Total	10152	3548

#### Biting midges

A total of 1701 biting midges, belonging to 16 species (Table 2), were trapped in 2017–2021. The most abundant species were *C. pictipennis* (*n* = 1,036, 61%), *C. festivipennis* (*n* = 203, 12%), *C. alazanicus* (*n* = 136, 8%) and *C. circumscriptus* (*n* = 136, 8%). Only 1% of trapped individuals (*n* = 18) were collected at Choteč, while the highest abundance of biting midges occurred in Zeměchy, with 1132 individuals (67%), the remaining 551 (32%) individuals being caught at Milovice forest.

**Table 2:** Species and numbers of *Culicoides* biting midges trapped in 2017–2021

Species	Total trapped	No. parous	No. of pools for analysis
*C. pictipennis*	1036	944	139
*C. festivipennis*	203	140	46
*C. alazanicus*	136	75	28
*C. circumscriptus*	136	11	31
*C. obsoletus*	88	40	36
*C. kibunensis*	26	23	9
*C. nubeculosus*	26	23	9
*C. segnis*	6	5	5
*C. vexans*	6	4	3
*C. maritimus*	3	3	1
*C. pallidocornis*	3	0	0
*C. duddingstoni*	2	0	0
*C. semimaculatus*	2	0	0
*C. clastrieri*	1	0	0
*C. punctatus*	1	1	1
*C. scoticus*	1	1	1
*C.* sp.	25	13	9
Total	1701	1283	318

Owing to the low number of caught individuals of *C. obsoletus* complex in other localities, we barcoded 20 randomly selected specimens from Milovice forest only, as from other sites only a few individuals [5] were caught. The barcoding revealed the presence of 19 *C. obsoletus* and 1 *C. scoticus*. Accordingly, we refer to these samples collectively as the *C. obsoletus* complex.

### Host feeding patterns according to blood meal detection

A total of 281 blood-engorged female mosquitoes were captured, and 185 of their blood meals (66%) were successfully barcoded. Only mammalian blood was detected in the mosquito genera *Aedes*, *Anopheles*, *Culiseta* and *Mansonia*. The most frequently identified mammalian blood source was deer blood, specifically from roe deer (*Capreolus capreolus*), red deer (*Cervus elaphus*) and fallow deer (*Dama dama*) (Table 3). Additionally, mammalian blood meals from seven sheep (*Ovis aries*), three European hares (*Lepus europeus*), three wild boar (*Sus scrofa*), two cattle (*Bos* sp.) and one field mouse (*Apodemus flavicollis*) were detected. In contrast, *Cx. pipiens* mosquitoes exhibited a broader host range, with the detection of avian, mammalian and amphibian blood. Specifically, the avian blood meals were obtained from species including five blackbirds (*Turdus merula*), two song thrushes (*Turdus philomelos*), one short-toed treecreeper (*Certhia brachydactyla*), one European robin (*Erithacus rubecula*), one great tit (*Parus major*) and one Eurasian blackcap (*Sylvia atricapilla*). The mammalian blood meal hosts included one sample positive for cattle (*Bos taurus*) and fallow deer (*D. dama)*, while an amphibian blood meal from a pool frog (*Rana lessonae*) was also detected. Human blood was detected across all mosquito genera, in 19 individuals total.

**Table 3:** Blood meals detected in mosquitoes and biting midges by barcoding

Insect species	Amphibian	Avian	Human	Non-human mammalian	Host species (number)	Total tested
*Ae. cantans/annulipes*	0	0	0	4	*Capreolus capreolus* (2) *Dama dama* (2)	5
*Ae. excrucians*	0	0	0	2	*Capreolus capreolus* (2)	2
*Ae. punctor*	0	0	0	2	*Cervus elaphus* (1) *Dama dama* (1)	3
*Ae. rusticus*	0	0	0	1	*Lepus europeus* (1)	1
*Ae.* sp.	0	0	1	7	*Dama dama* (4) *Capreolus capreolus* (2) *Cervus elaphus* (1) *Homo sapiens* (1)	10
*Ae. vexans*	0	0	7	67	*Cervus elaphus* (25) *Dama dama* (23) *Capreolus capreolus* (12) *Homo sapiens* (7) *Ovis aries* (4) *Lepus europeus* (2) *Bos frontalis* (1)	116
*An. maculipennis*	0	0	0	9	*Dama dama* (5) *Cervus elaphus* (4)	11
*An. plumbeus*	0	0	1	2	*Apodemus flavicollis* (1) *Dama dama* (1) *Homo sapiens* (1)	5
*Cx. pipiens*	1	11	8	2	*Homo sapiens* (8) *Turdus merula* (5) *Turdus philomelos* (2) *Certhia brachydactyla* (1) *Erithacus rubecula* (1) *Parus major* (1) *Sylvia atricapilla* (1) *Rana lessonae* (1) *Bos taurus* (1) *Dama dama* (1)	42
*Cs. annulata*	0	0	1	40	*Dama dama* (20) *Cervus elaphus* (15) *Ovis aries* (3) *Capreolus capreolus* (1) *Homo sapiens* (1) *Sus scrofa* (1)	61
*Ms. richiardii*	0	0	1	18	*Dama dama* (10) *Cervus elaphus* (5) *Sus scrofa* (2) *Capreolus capreolus* (1) *Homo sapiens* (1)	23
*C. pictipennis*	0	0	13	0	*Homo sapiens* (13)	37
Total	1	11	32	154		318

Furthermore, 52 blood-engorged biting midges were collected, all belonging to the species *C. pictipennis*, and 13 of their blood meals (25%) were successfully barcoded. All identified blood meals contained exclusively human blood.

### Prevalence of parasites in the insects

#### Mosquitoes

In total, 7905 mosquitoes were divided into 1298 (i.e. average pool size: 7 individuals) pools and tested for haemosporidians and trypanosomes using PCR. Additionally, 2246 individual mosquitoes were examined by dissection. Parasites were detected in five mosquito genera (Table 4), in nearly all identified mosquito species except for *Ae. caspius*, *Ae. communis*, *Ae. flavescens*, *Ae. rusticus* and *Cs. morsitans*.

**Table 4:** Overall parasite diversity and MIR found in mosquitoes in 2017–2021

	Total		Haemosporidians							Avian trypanosomes							Mammalian trypanosomes		
Mosquito species	No. of individuals	No. of pools	No. of positive pools	MIR	No. of *P. matutinum*	No. of *P. relictum*	No. of *P. vaughani*	No. of *P.* sp.	No. of *H*. sp.	No. of positive pools	MIR	*T. avium*	*T. culicavium*	*T. tertium*	*T. thomasbancrofti*	*T.* sp. from group B	No. of positive pools	MIR	*T. theileri*
*AEDES*	2543	1318	1	0.04%	1	–	–	–	–	2	0.08%	–	1	–	–	1	227	9%	227
*Ae. vexans*	1877	1060	1	0.05%	1	–	–	–	–	1	0.05%	–	–	–	–	1	176	9%	176
*Ae.* sp.	276	83	–	–	–	–	–	–	–	–	–	–	–	–	–	–	15	5%	15
*Ae. annulipes/cantans*	113	47	–	–	–	–	–	–	–	–	–	–	–	–	–	–	15	13%	15
*Ae. punctor*	89	49	–	–	–	–	–	–	–	1	1%	–	1	–	–	–	12	13%	12
*Ae. cinereus*	76	15	–	–	–	–	–	–	–	–	–	–	–	–	–	–	1	1%	1
*Ae. excrucians*	38	24	–	–	–	–	–	–	–	–	–	–	–	–	–	–	5	13%	5
*Ae. sticticus*	7	7	–	–	–	–	–	–	–	–	–	–	–	–	–	–	2	29%	2
*Ae. cataphylla*	2	2	–	–	–	–	–	–	–	–	–	–	–	–	–	–	1	50%	1
*ANOPHELES*	139	89	–	–	–	–	–	–	–	2	1%	1	1	–	–	–	6	4%	6
*An. plumbeus*	83	54	–	–	–	–	–	–	–	–	–	–	–	–	–	–	2	2%	2
*An. maculipennis*	32	25	–	–	–	–	–	–	–	1	3%	1	–	–	–	–	1	3%	1
*An. claviger*	24	10	–	–	–	–	–	–	–	1	4%	–	1	–	–	–	3	13%	3
*CULEX*	6747	1811	123	2%	–	–	–	–	–	89	1%	1	80	2	3	3	5	0.07%	5
*Cx. pipiens*	6729	1796	121	2%	63	46	6	3	3	88	1%	1	79	2	3	3	5	0.07%	5
*Cx. modestus*	14	11	2	14%	2	–	–	–	–	1	7%	–	1	–	–	–	–	–	–
*CULISETA*	228	152	1	0.4%	–	–	–	–	–	1	0.4%	1	–	–	–	–	2	1%	2
*Cs. annulata*	215	147	1	0.5%	1	–	–	–	–	1	0.5%	1	–	–	–	–	2	1%	2
*MANSONIA*	495	178	2	0.4%	–	–	–	–	–	2	0.4%	–	2	–	–	–	4	1%	4
*Ms. richiardii*	495	178	2	0.4%	2	–	–	–	–	2	0.4%	–	2	–	–	–	4	1%	4

Haemosporidian parasites were predominantly found in *Culex* mosquitoes (no. of positive pools = 124, MIR = 2%). The most prevalent haemosporidian in *Cx. pipiens* was *P. matutinum* (*n* = 64), with the LINN1 lineage being the most common, detected in 61 samples (Table 5). Additionally, two AFTRU5 and one TUPHI08 lineages were also detected. In *Cx. modestus*, the lineage LINN1 and an unresolved LINN1/TURMER09 lineage (due to short sequence length) were also detected. *P. matutinum* was the only species detected in mosquito genera other than *Cx. pipiens*. Lineage LINN1 was identified in one specimen each of *Ae. vexans*, *Cs. annulata* and *Ms. richiardii*. A novel *P. matutinum* lineage, MANSON03 (GenBank accession no. PV085844), was detected in *Ms. richiardii*.

**Table 5:** Haemosporidian lineages identified in mosquitoes and biting midges

Species	Lineage	*n*	Detected in	Method	Country
*H. asymmetricus*/*H. minutus* or *H*. sp.	SYCUR01^a^ or TUPHI01^a^	7	*C. pictipennis*	PCR	TUPHI01: AUT, BGR, CAN, CHE, CZE, DEU, DNK, GBR, LTU, PER, PRT, RUS, SRB, SVK, SWE SYCUR01: ARM
		1	*C. kibunensis*	PCR	
		1	*C. nubeculosus*	PCR	
		1	*Cx. pipiens*	PCR	
*H. concavocentralis*	HAWF2^a^	2	*C. pictipennis*	PCR	BGR, SVK
*H. belopolskyi*	MW1^a^	1	*Cx. pipiens*	PCR	BGR, ESP, IND, KEN, MWI, NGA, NLD, PRT, RUS, SRB, SWE, TUR
*H.* sp.	HAWF6^a^	1	*C. pictipennis*	PCR	AUT, DEU, MOR, PRT, SVK
*P. matutinum*	LINN1	61	*Cx. pipiens*	PCR (47), dissected (14)	AUT, CHE, CZE, DEU, ESP, FIN, FRA, GBR, HUN, ITA, JPN, NZL, POL, PRT, SVK, SWE, USA
		1	*Cx. modestus*	PCR	
		1	*Ms. richiardii*	PCR	
		1	*Cs. annulata*	dissected	
		1	*Ae. vexans*	dissected	
		1	*C. kibunensis*	PCR	
	AFTRU5^a^	2	*Cx. pipiens*	PCR	AUT, CMR, DEU, ESP, GNQ, IND, ISR, ITA, MOR, NGA, NZL, POL, PRT, SWE, USA
	LINN1 or TURMER09^a^	1	*Cx. modestus*	PCR	LINN1: See above TUMER09: SVK
	TUPHI08^a^	1	*Cx. pipiens*	PCR	AUT, SVK
	MANSON03^b^	1	*Ms. richiardii*	PCR	CZE
*P. relictum*	SGS1	25	*Cx. pipiens*	PCR	ARM, AUT, BEL, BEN, BGR, CAN, CHE, CHN, CZE, DEU, DZA, EGY, ESP, FIN, FLK, FRA, GBR, GRC, HUN, IND, ISR, ITA, JPN, KEN, KOR, LTU, MNG. MOR, NGA, NLD, NOR, NZL, PER, POL, PRT, ROU, RUS, SRB, SVK, SWE, TUN, TUR, UKR, USA, ZAF
	SYCON02^a^	4	*Cx. pipiens*	PCR	ESP
	ALERUF05^a^	3	*Cx. pipiens*	PCR	ESP
	GRW11	2	*Cx. pipiens*	PCR	ARM, AUT, BGR, CZE, CHE, DEU, DZA, ESP, FRA, GBR, HUN, ISR, ITA, JPN, LTU, MOR, NGA, NLD, POL, PRT, ROU, RUS, SRB, SVK, SWE, TUN, TUR, UKR, ZAF
	SGS1 or PARUS75	1	*Cx. pipiens*	dissected	SGS1: See above PARUS75: MOR
	CXPIP23^a^	1	*Cx. pipiens*	PCR	BGR, TUR
	CXPIP40^b^	1	*Cx. pipiens*	PCR	CZE
	CXPIP41^b^	1	*Cx. pipiens*	PCR	CZE
	CXPIP42^b^	1	*Cx. pipiens*	PCR	CZE
*P. vaughani*	SYAT05^a^	6	*Cx. pipiens*	PCR (3), dissected (3)	ARM, AUT, BRA, BGR, CHE, CHN, DEU, ESP, GAB, HUN, ITA, IRN, JPN, KEN, MOR, NLD, NZL, PRT, RUS, SRB, SVK, SWE, USA
*P. elongatum*	GRW06	1	*Cx. pipiens*	PCR	AUS, AUT, BIH, BRA, BGR, CHE, CHN, CMR, COL, CPV, CZE, DEU, ESP, FRA, GAB, GLP, IND, ITA, JPN, MDG, MMR, MNG, MWI, NIC, NZL, PNG, POL, PRT, RUS, SRB, STP, SVK, SWE, THA, TUN, TUR, TZA, USA, VEN, ZAF, ZMB
*P.* sp.	COLL1^a^	1	*Cx. pipiens*	dissected	AUT, BGR, ESP, FRA, POL, PRT, ROU, SVK, TUN, ZAF
	TUMER05^a^	1	*Ms. richiardii*	PCR	MOR

This table presents data only for quality sequences that could be used for lineage determination. Country abbervations: ARM,Armenia; AUS, Australia; AUT, Austria; BEL, Belgium; BEN, Benin; BGR, Bulgaria; BIH, Bosnia and Herzegovina; BRA, Brazil; CAN, Canada; CHE, Switzerland; CHN, China; CMR, Cameroon; COL, Colombia; CPV, Cabo Verde; CZE, Czechia; DEU, Germany; DNK, Denmark; DZA, Algeria; EGY, Egypt; ESP, Spain; FIN, Finland; FLK, Falkland Islands (Malvinas); FRA, France; GAB, Gabon; GBR, United Kingdom; GLP, Guadeloupe; GNQ, Equatorial Guinea; GRC, Greece; HUN, Hungary; IND, India; IRN, Iran; ISR, Israel; ITA, Italy; JPN, Japan; KEN, Kenya; KOR, South Korea; LTU, Lithuania; MDG, Madagascar; MMR, Myanmar; MNG, Mongolia; MOR, Morocco, MWI, Malawi; NGA, Nigeria; NIC, Nicaragua; NLD, Netherlands; NOR, Norway; NZL, New Zealand; PER, Peru; PNG, Papua New Guinea; POL, Poland; PRT, Portugal; ROU, Romania; RUS, Russia; SRB, Serbia; STP, São Tomé and Príncipe; SVK, Slovakia; SWE, Sweden; THA, Thailand; TUN, Tunisia; TUR, Türkiye; TZA, Tanzania; UKR, Ukraine; USA, United States; VEN, Venezuela; ZAF, South Africa; ZMB, Zambia.

^a^New finding for Czechia

^b^Novel lineages found in this study

The second most prevalent haemosporidian species detected was *P. relictum*, found exclusively in 46 specimens of *Cx. pipiens*. Of these, 38 were successfully barcoded, revealing five known lineages and three novel lineages; the remaining eight samples could not be assigned to a lineage owing to unsuccessful or poor-quality sequencing. The most abundant *P. relictum* lineages identified were SGS1 (*n* = 25), followed by four SYCON02, three ALERUF05, two GRW11 and one CXPIP23 lineages. Novel lineages were designated as CXPIP40 (accession no. PV085841), CXPIP41 (accession no. PV085842) and CXPIP42 (accession no. PV085843).

Only one lineage of *P. vaughani*, SYAT05, was detected in six *Cx. pipiens* specimens, along with one GRW06 lineage of *P. elongatum* and the COLL1 lineage of *Plasmodium* sp.

Oocysts were observed in 68 dissected individuals across several mosquito species: *Culex pipiens* (*n* = 48), *Ae. vexans* (*n* = 18), *Cs. annulata* (*n* = 2), *Ms. richiardii* (*n* = 2), *Ae. caspius* (*n* = 1) and *An. plumbeus* (*n* = 1). However, only 24 (35%) of these were successfully barcoded, revealing lineages of 14 *P. matutinum* LINN1, three *P. relictum* SYCON02, three *P. matutinum* SYAT05 and one *P.* sp. COLL1 in *Cx. pipiens*. The LINN1 lineage of *P. matutinum* was identified also in the dissected *Ae. vexans* and *Cs. annulata* specimens that displayed oocysts with sporozoites (Table 5).

*Haemoproteus* was detected only in *Cx. pipiens* specimens, specifically the lineage MW1 (*H. belopolskyi*) and an unspecified lineage closely related to SYCUR01 or TUPHI01 (*H. asymetricus*/*H. minutus* or *H*. sp.), which could not be determined more precisely owing to short sequence length (Table 5). In total, 13 haemosporidian-positive pools could not be identified to lineage level owing to short or low-quality sequences. *Leucocytozoon* was not detected in any of the mosquito pools.

Avian trypanosomes were detected in 90 positive specimens with a minimum infection rate of 1% (Table 4). The most abundant avian trypanosome species was *T. culicavium*, identified in 84 mosquitoes, specifically in 79 samples of *Cx. pipiens*, 2 *Ms. richiardii* and 1 each of *Ae. punctor*, *An. claviger* and *Cx. modestus*. Additional avian trypanosome species detected included *T. thomasbancrofti* (*n* = 3) and *T. tertium* (*n* = 2), all found in *Culex pipiens* mosquitoes. Furthermore, *T.* sp. from group B (*n* = 4) was detected in three *Cx. pipiens* and one *Ae. vexans*. All *T.* sp. had a 100% match with isolate PAS95. In contrast, *T. avium* was identified in three different mosquito species, i.e. *An. maculipennis* (*n* = 1), *Cs. annulata* (*n* = 1) and *Cx. pipiens* (*n* = 1).

Several co-infections of *T. culicavium* and haemosporidians were detected in individually examined *Cx. pipiens* specimens. These trypanosomes co-occurred with *P. matutinum* (LINN1) twice, once with *P. vaughani* (SYAT05) and once with *Haemoproteus* sp. (SYCUR01 or TUPHI05).

Mammalian trypanosomes of the *T. theileri* group were found in all mosquito genera, but predominantly in *Aedes* mosquitoes, with a prevalence of 9% (*n* = 227) across seven species: *Ae. vexans* (*n* = 176), *Ae. cantans*/*annulipes* (*n* = 15), *Ae. punctor* (*n* = 12), *Ae. excrucians* (*n* = 5), *Ae. sticticus* (*n* = 2), *Ae. cataphylla* (*n* = 1) and *Ae. cinereus* (*n* = 1). Additionally, *T. theileri* was detected in five *Cx. pipiens*, four *Ms. richiardii*, three *An. claviger*, two *An. plumbeus*, two *Cs. annulata* and one *An. maculipennis* (Table 4).

**Fig. 2:**
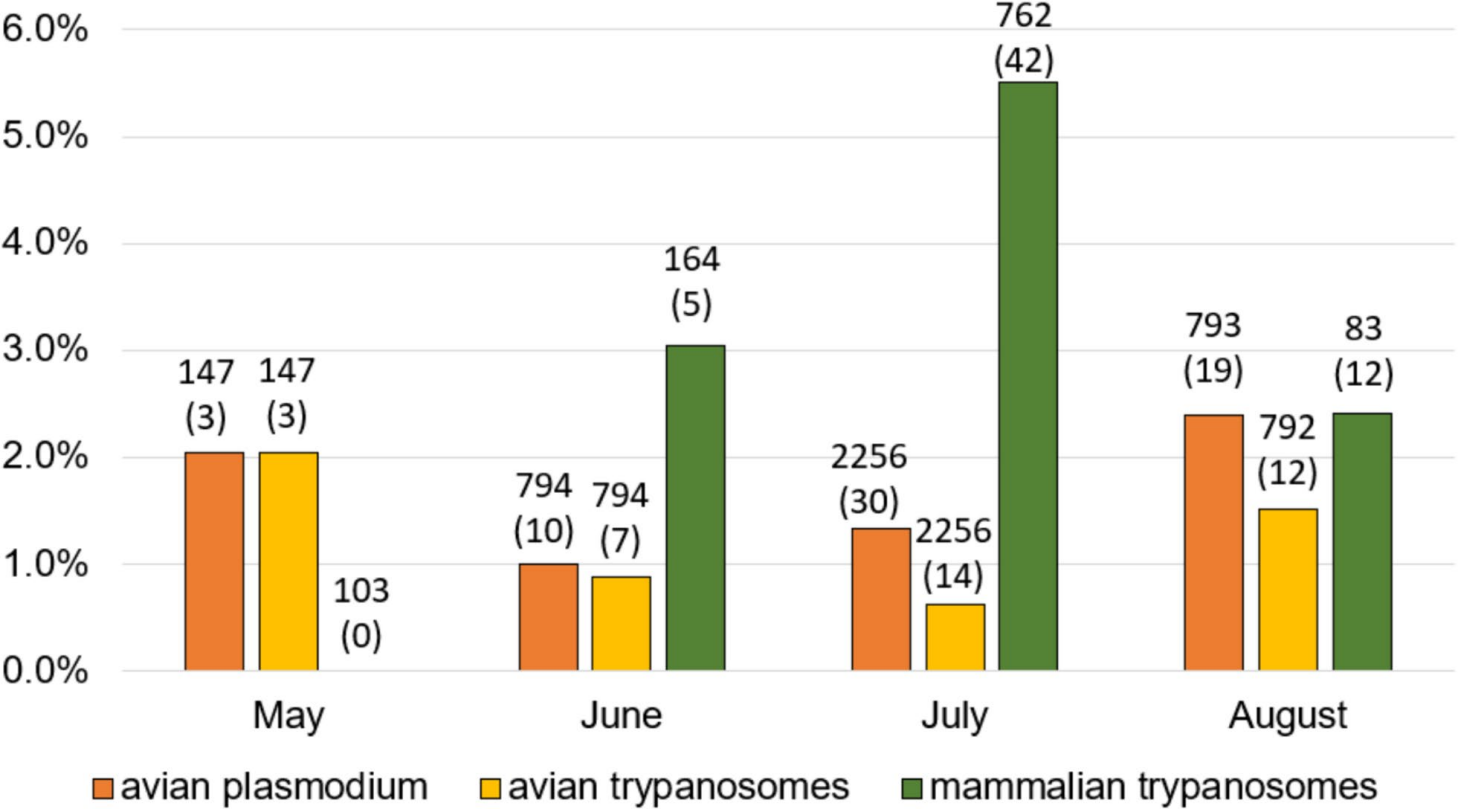
Prevalences of parasites (counted as MIR) in mosquitoes according to month of trapping. Years and localities are merged. *Plasmodium* and avian trypanosomes refer to *Culex* spp. mosquitoes, mammalian trypanosomes to *Aedes* spp. Numbers above columns represent the number of positive pools; numbers in brackets represent caught individuals

*Plasmodium* was detected in *Culex* mosquitoes throughout the 4-month monitoring period. An initial prevalence of 2% was observed at the beginning of the season, declining to 1% in June, and then increasing again to a seasonal maximum of 2.4% in August. Avian trypanosomes were consistently detected in *Cx. pipiens* throughout the study period, with relatively stable prevalence ranging from 0.6% to 2% (Fig. 2). In contrast, mammalian trypanosomes showed a different pattern: they were absent in *Aedes* mosquitoes in May, rose to 3% in June, peaked at 5.5% in July and declined to 2.4% in August.

#### Biting midges

Overall, 1283 parous or dark biting midges (75% of caught midges) were divided into 318 pools and examined by PCR for the presence of blood parasites (Table 6). The analysis revealed three distinct genera of blood parasites in these insects: *Plasmodium*, *Haemoproteus* and *Trypanosoma*.

**Table 6:** Overall parasite diversity and MIR found in biting midges in 2017–2021

Biting midge species	Total		Haemosporidians				Avian trypanosomes		
	No. of parous	No. of pools	No. of positive pools	MIR	No. of *P. matutinum*	No. of *H*. sp.	No. of positive pools	MIR	No. of *T. bennetti* s.l.
*C. pictipennis*	944	139	10	1%	–	10	4	0.4%	4
*C. festivipennis*	140	46	–	–	–	–	1	1%	1
*C. alazanicus*	75	28	–	–	–	–	1	1%	1
*C. kibunensis*	23	9	2	9%	1	1	2	9%	2
*C. nubeculosus*	23	9	1	4%	–	1	–	–	–

Specifically, *P. matutinum* lineage LINN1 was identified in a single specimen of *C. kibunensis*. Additionally, the HAWF2 lineage of *H. concavocentralis* and the HAWF6 lineage of *Haemoproteus* sp. were identified in *C. pictipennis*. Furthermore, *H. asymetricus/H. minutus* or *Haemoproteus* sp. (lineage SYCUR01 or TUPHI01) was detected in *C. pictipennis*, *C. kibunensis* and *C. nubeculosus* (Table 5).

Avian trypanosomes of the *T. bennetti* group (*n* = 8), including lineages VI, VIII and Cfest (relating to VIII), were found in four biting midge species: *C. pictipennis*, *C. kibunensis*, *C. alazanicus* and *C. festivipennis* (Table 6). In addition, a co-infection of *T. bennetti* (lineage VIII) and *Haemoproteus* sp. (SYCUR01 or TUPHI01) was observed in one specimen of *C. kibunensis*.

### Comparison of the two methods

**Fig. 3:**
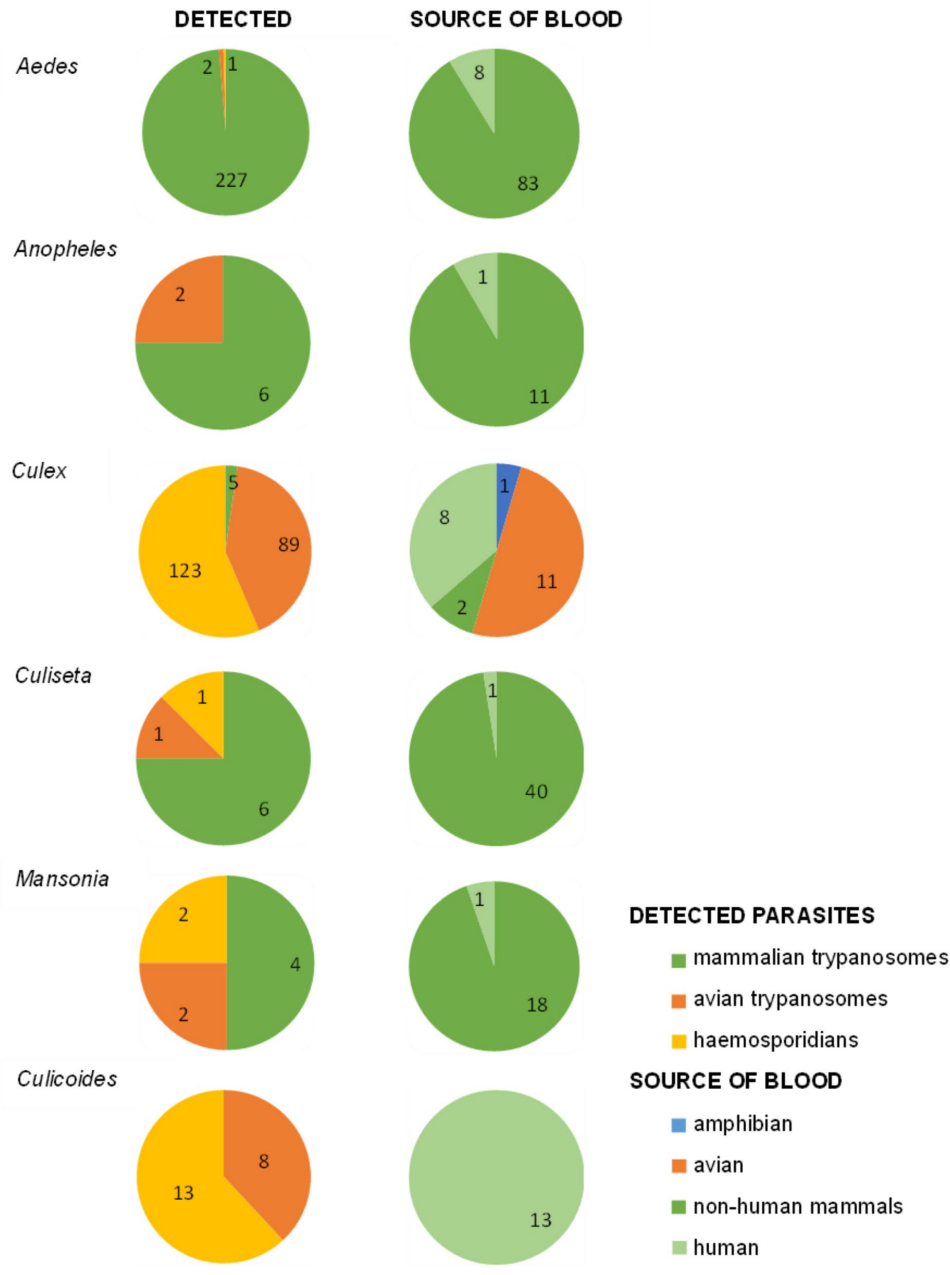
Source of blood (B) and parasites (P) detected in mosquitoes and midges

In four mosquito genera—*Aedes*, *Anopheles*, *Culiseta* and *Mansonia*—blood meal analysis revealed exclusively mammalian blood (Fig. 3). Moreover, the majority of trypanosomes detected in *Aedes* mosquitoes were mammalian, with only 1% (*n* = 3) being avian parasites. For the genera *Anopheles*, *Culiseta* and *Mansonia*, parasite detection revealed both avian and mammalian parasites. Specifically, two *Anopheles* samples were positive for avian trypanosomes, and six samples were positive for mammalian trypanosomes. In *Culiseta*, one sample was positive for *Plasmodium*, and one for an avian trypanosome, while mammalian trypanosomes were detected in six samples. For *Mansonia*, half of the detected parasites were mammalian trypanosomes, and half were avian, including two *Plasmodium* and two avian trypanosome infections.

*Culex* mosquitoes exhibited diverse feeding patterns, feeding on avian (*n* = 11), mammalian (*n* = 10, including eight human samples) and amphibian (*n* = 1) hosts. This resulted in a relatively even distribution of mammalian and avian blood sources. In contrast, parasites detected in *Culex* mosquitoes were predominantly of avian origin, with 123 pools positive for haemosporidians and 90 for avian trypanosomes, while mammalian trypanosomes accounted for only 2% of detected parasites.

Screening of blood-fed mosquitoes for parasites revealed several noteworthy host–parasite combinations. *T. theileri*, a mammalian trypanosome, was detected in nine *Aedes* individuals that had fed on cervids and in one individual that had fed on a human. *T. culicavium*, an avian trypanosome, was detected in one *Cx. pipiens* mosquito containing human blood. Additionally, human blood was identified in dissected *Ae. vexans* mosquitoes that also harboured *P. matutinum* oocysts in the gut. Furthermore, simultaneous infections of *T. culicavium* and *Haemoproteus* sp. (TUPHI01 or SYCUR01) were detected in two additional *Cx. pipiens* mosquitoes that had fed on the song thrush (*Turdus philomelos*). Another *Cx. pipiens* individual with song thrush blood was also positive for *Haemoproteus* sp. (lineage unidentified owing to low-quality sequence). Moreover, a *Cs. annulata* mosquito feeding on fallow deer (*D. dama*) blood was found to be infected with *T. theileri*.

Human blood meals were exclusively detected in 13 specimens of the biting midge species *C. pictipennis*. Conversely, parasitological examination revealed only avian parasites in this species as well as in other biting midges. Notably, the avian parasite *H. concavocentralis* (lineage HWF2) was identified in one *C. pictipennis* individual that had fed on human blood.

## Discussion

### Vector composition and species prevalence

The study identified 19 mosquito species out of 45 recorded in Czechia [27], reflecting a broad spectrum that is consistent with previous findings [28, 29] with *Culex pipiens* (66%) being dominant, followed by *Aedes vexans* (18%). *Culex modestus*, a key vector of West Nile and Usutu viruses, has established populations in Czechia, mainly in wetlands [30, 31]. That corresponds with the highest number of trapped individuals in Zeměchy, a site characterised by reed beds. However, the prevalence was not as high as in other wetland areas in Czechia [2]. Its lower numbers likely reflect dry summer conditions (dry water source), as population peaks typically occur in July–August [28].

We identified 15 of the 49 *Culicoides* species known from Czechia [32], with *C. pictipennis* (61%) and *C. festivipennis* (12%) as dominant species. This contrasts with the work of Rádrová et al. (2016) [33], where *C. obsoletus* dominated, likely owing to differences in attractants (CO_2_-baited traps versus UV light traps) and trapping sites (forest versus stall), which can influence species composition [34, 35]. Barcoding of the *C. obsoletus* complex revealed that the majority of the individuals belonged to the species *C. obsoletus*, with a minor presence of *C. scoticus*.

### Feeding patterns from blood meal barcoding

Blood meal analysis showed that *Culex* mosquitoes feed on a broad range of hosts, birds (50%), mammals (45%) and amphibians (5%), indicating an opportunistic feeding strategy. This aligns with previous studies reporting 60–82% of avian blood in fed *Cx. pipiens* across Europe [36–40]. Amphibian feeding, previously documented in *Cx. pipiens* [2, 41, 42], appears to be occasional and likely incidental, on the basis of our findings (5%).

*Aedes* mosquitoes are generally considered mammalophilic or slightly opportunistic [43, 44], which corresponds with our findings. In our study, *Aedes* mosquitoes primarily fed on large cervids, the most common hosts not only at Milovice forest, which is a game reserve, but also at other trapping sites. *Ae. vexans* exhibited the widest host range, feeding on seven mammal species including *C. elaphus*, *C. capreolus* and *D. dama*, and also humans (9%), similarly to previous studies [39, 43, 45]. Other *Aedes* species, such as *Ae. cantans*/*annulipes*, *Ae. excrucians* and *Ae. punctor*, also fed mainly on deer, consistent with earlier findings [39, 44].

*Anopheles*, *Mansonia* and *Culiseta* mosquitoes fed exclusively or predominantly on mammals [11, 46–51]. In our study, *Anopheles* species such as *An. maculipennis* and *An. plumbeus* fed exclusively on mammals, including humans, a pattern that supports their known mammalophilic behaviour and role in human malaria transmission [11, 47, 51]. Only mammalian blood was also detected in *Ms. richiardii*, supporting its general classification as mammalophilic [49], although occasional avian feeding has been reported under certain conditions [52]. Mosquitoes of genus *Culiseta* are generally considered opportunistic feeders with a propensity for avian hosts [46, 48], however all blood meals we detected in our samples were from mammals, likely reflecting local host availability or sampling bias.

Among *Culicoides*, only *C. pictipennis* was found blood-fed, and all blood meals originated from humans. However, considered an opportunistic feeder [53, 54], nearly all positives were from a single trapping event at a site with known presence of homeless people, suggesting opportunistic use of a locally abundant host.

### Inferring feeding patterns and vector potential through parasite detection

A total of 10,152 female mosquitoes, representing five genera (*Aedes*, *Anopheles*, *Culex*, *Culiseta* and *Mansonia*) were screened for haemosporidians and trypanosomes. Haemosporidians were detected in 1.2% of the mosquitoes, most frequently in *Culex* (2%), consistent with earlier reports ranging from 0.8% to 4.3%, although higher values (up to 5.3%) have been observed in some European wetlands [55–59]. Avian trypanosomes were found in 0.94% of the mosquitoes overall, again most commonly in *Culex* (1.3%), with *T. culicavium* as the predominant species, consistent with earlier studies [60–63]. Mammalian trypanosomes of the *T. theileri* group were detected in 2.4% of specimens, with the highest prevalence in *Aedes*, a genus regarded as a potential vector [23], They were also present in other primarily mammalophilic genera, including *Anopheles*, *Culiseta* and *Mansonia*.

*Culex pipiens* hosted the highest diversity of *Plasmodium* spp., with 14 lineages detected, highlighting the significant role of this mosquito as a vector of avian malaria in Europe [55, 64]. Most of the detected parasites belonged to three common lineages: *P. matutinum* (LINN1), *P. relictum* (SGS1) and *P. vaughani* (SYAT05), all previously reported across Europe [55, 65–67]. Our study also revealed seven *Plasmodium* lineages not previously reported in Czechia, all found in *Cx. pipiens*, and one additional lineage in *Cx. modestus*. Three distinct *P. relictum* lineages (CXPIP40, CXPIP41 and CXPIP42) were each found in single *Cx. pipiens* individuals, further expanding the known diversity of avian haemosporidians.

In addition to avian haemosporidians, trypanosomes were also detected in *Culex* mosquitoes. Besides the predominantly occurring avian *T. culicavium*, we identified *T. thomasbancrofti* and *T. tertium* as well, both with low prevalence (0.04% and 0.02%, respectively), similar to previous studies [63, 68]. The detection of *T. theileri* in *Cx. pipiens* (5/6747), a mosquito species not considered a competent vector [23], suggests occasional feeding on mammals and highlights the value of parasite data for inferring host feeding patterns.

The parasite composition reflected the feeding behaviour of *Cx. pipiens*: 98% of infections were avian (58% haemosporidians, 42% trypanosomes) and only 2% were mammalian, mirroring the blood meal results and supporting an overall opportunistic feeding strategy. This is consistent with behavioural studies, in which *Cx. pipiens* showed no strong preference among bird, mouse and human hosts [69]. Although *Cx. pipiens* biotypes were not distinguished in our study, the ornithophilic *pipiens* form likely dominated in our rural sites, while the *molestus* form, which is more mammalophilic, typically occurs in urban areas [70]. Feeding patterns in *Cx. pipiens* are influenced by both genetic background and local host availability [70–72].

In addition to *Plasmodium*, *Cx. pipiens* also carried *Haemoproteus* lineages MW1 and SYCUR01/TUPHI1. *Haemoproteus* has already been reported in *Aedes*, *Culex*, *Coquillettidia* and *Mansonia*, but their vector competence remains unconfirmed [73–75]. Notably, in experiments with *Cx. pipiens*, no parasite DNA in salivary glands was detected after feeding on infected birds [57]. Similarly in *Ae. cantans*, oocysts formed without sporozoites, indicating abortive development. However non-infective parasite stages may still be detectable by PCR for several weeks [14, 15].

Mammalian trypanosomes of the *T. theileri* group were predominant in *Aedes* mosquitoes, accounting for 98.5% of the detected parasites, in line with our previous findings indicating *Aedes* as a probable vector species [23]. In contrast, only three samples contained avian parasites, namely *T. culicavium*, *Trypanosoma* sp. from group B and *P. matutinum*. While *Aedes* mosquitoes can harbour detectable parasite DNA, recent experiments have shown that the ability of avian trypanosomes to develop infective stages in *Aedes* is negligible or absent [68, 76]. However, certain avian *Plasmodium* species are able to complete their development in *Aedes* mosquitoes [77]. Our study revealed the presence of oocysts containing sporozoites in the dissected midgut of two *Ae. vexans* mosquitoes. In one case, the parasite was identified as the LINN1 lineage of *P. matutinum*, while the second oocyst-positive sample could not be identified because it tested negative by PCR.

Despite dominantly detected mammalian blood meals in *Culiseta*, *Mansonia* and *Anopheles*, avian parasites were frequently detected. In *Ms. richiardii*, half of the detected parasites were avian. *Ms. richiardii* carried avian *P. matutinum* (LINN1), and we also identified a novel lineage, MANSON03, in coinfection with TUMER05 previously reported in Morocco [78]. Furthermore, *Plasmodium* oocysts were also observed in two dissected individuals, though these were not successfully barcoded. Similar findings of sporozoites in *Mansonia* have been reported previously, suggesting that they may play a role in transmitting certain plasmodium lineages [58, 73, 79]. Notably, a study in Lithuania reported an avian plasmodium prevalence of 2.5% in *Ms. richiardii*, higher than the 0.4% prevalence we observed [58]. We also detected avian *T. culicavium* in two samples (0.4%), supporting occasional bird feeding in this species. In addition, *P. matutinum* (LINN1) oocysts were also detected in dissected *Cs. annulata*. Previous studies have reported the presence of *Plasmodium* DNA in the head and thorax of *Culiseta*, together indicating potential vector competence [58, 80]. These findings expand the known diversity and host range of avian *Plasmodium* and *Trypanosoma* in Central European mosquitoes.

The *Anopheles* genus is predominantly mammalophilic and is notoriously known for transmitting *Plasmodium* to humans [11, 47, 51]. This finding is consistent with our blood meal analysis, which showed that *Anopheles* mosquitoes fed exclusively on mammalian hosts, including humans. Nevertheless, we did not detect any haemosporidian parasites in this genus, but 25% of identified parasites were avian trypanosomes, indicating that *Anopheles* mosquitoes have fed on birds. This likely reflects occasional avian feeding in mammal-dominated habitats [81]. The remaining parasites belonged to mammalian trypanosomes of the *T. theileri* group.

In *Culicoides*, only avian parasites were detected, in contrast to the blood meal analysis. Biting midge specimens were were infected with haemosporidian parasites at the minimum infection rate of 1.1%, which is notably lower than previously reported prevalence rates of 5.2% for *Haemoproteus* and 7.9% for *Plasmodium* and *Haemoproteus* in biting midges [82, 83]. Three *Haemoproteus* lineages were detected in *C. kibunensis*, *C. nubeculosus* and *C. pictipennis*, with TUPHI01/SYCUR01 being the most frequent. TUPHI01, *H. asymmetricus*/*H. minutus*, had been previously reported in *Culicoides* from Czechia and Lithuania [65, 84], whereas SYCUR01 had only been reported from Armenia (MalAvi database). *P. matutinum*, commonly described in *Culex* mosquitoes [58, 85, 86] and also detected in *C. pictipennis* [87], was found in one *C. kibunensis* pool in our study. In addition to haemosporidians, we detected several avian *Trypanosoma* lineages in eight *Culicoides* pools, lineages VI, VIII and Cfest115—belonging to the *T. bennetti*/*everetti* group, all previously detected in Czechia [88, 89]. Despite feeding exclusively on humans in our samples, *C. pictipennis* tested positive for avian parasites, confirming its opportunistic feeding habits as reported earlier [53, 54].

Parasites do not always have a monopoly within their vectors and are frequently found in co-infection with other parasites. We detected co-infections in three individually examined *Cx. pipiens* mosquitoes, involving *T. culicavium* and either *Haemoproteus* (TUPHI01/SYCUR01), *P. matutinum* (LINN1) or *P. vaughani* (SYAT05). A further co-infection was found in *C. kibunensis*, with *Haemoproteus* and *T. bennetti* (lineage VIII). In the case of trypanosomes, the co-infection insights remain poorly understood. However, recent experiments with *Anopheles* mosquitoes have shown that exposure to *Trypanosoma* may increase susceptibility to *Plasmodium* infection [90].

### Seasonal dynamics of parasite prevalence

Avian *Plasmodium* prevalence typically increases over the season, as observed in Austria, Spain, Switzerland and Lithuania [44, 67, 91, 92]. Similarly, avian trypanosome prevalence has been shown to peak in August, in line with observations from Austria [60]. In our study, both parasite groups showed a mid-season decline (June–July), followed by a late-season peak. This pattern may be explained by the emergence of new, uninfected mosquito generations, which can occur three to four times per year [17], or by interannual variation in temperature and rainfall [93].

The absence of avian parasites in overwintering *Cx. pipiens* may also contribute to low prevalence observed at the beginning of the season [92]. Mammalian *T. theileri* was not detected in May, peaked in July and declined in August, following a seasonal trend similar to previous findings in Austria [60].

### Comparison of blood barcoding versus parasite detection

To assess host–parasite interactions in more detail, we compared two complementary approaches: blood meal barcoding and parasite detection, on a total of 10,152 female mosquitoes, which were screened for haemosporidian and trypanosome infection; 281 of those were blood-fed, with 185 (66%) successfully barcoded. This blood barcoding efficiency falls within the typical range of 44–72% (occasionally reaching 90% or 99%), and largely depends on the digestion stage of the blood meal [41, 56, 94–97]. In *Culicoides*, only 37 engorged individuals were captured, with a 25% success rate, which is consistent with previous reports showing wide variability (29–92%) depending on blood meal digestion [53, 98–100]. The low barcoding success observed in our study is likely due to partial digestion of the blood meal. The low number of engorged individuals likely reflects the use of CO_2_-baited traps, which primarily attract host-seeking rather than blood-fed insects. To collect more blood-fed vectors, supplementing trapping with methods targeting resting individuals, such as aspiration from shelters, indoor collections or resting boxes, would be beneficial. These approaches would increase the number of blood-fed insects and enhance the interpretative power of blood meal analysis.

Parasite detection successfully identified 469 positive mosquito samples (2.5 times more than the number of successfully barcoded blood meals) from 14 different mosquito species. The overall parasite prevalence across these samples was 4.6%. A similar trend was observed in *Anopheles*, where parasite-positive specimens outnumbered those with identifiable blood meals tenfold [11]; a similar pattern was observed in Ref. [56], despite a higher PCR success rate. However, this trend may reverse in studies targeting a specific pathogen. For example, in an analysis focused on *Theileria orientalis*, 99% of blood meal samples were successfully identified, while only 15.2% yielded parasite DNA [95]. This contrast reflects the broader scope of our screening strategy, which targeted multiple protozoan parasites rather than a single species.

While parasite detection offers greater sensitivity in terms of infection prevalence, blood barcoding provides more precise host identification, especially relevant for assessing potential transmission to humans. In our region, where no human-infecting protozoan vector-borne parasites are currently circulating, the ability of barcoding to detect human blood meals remains particularly valuable. Conversely, parasite detection is less host-specific but contributes crucial data on infection rates and vector–parasite compatibility.

Importantly, the two methods can yield contrasting but complementary insights. For example, barcoding suggested that *An. maculipennis*, *Ms. richiardii*, *Cs. annulata*, *Ae. punctor*, *Ae. vexans* and *C. pictipennis* were predominantly mammalophilic, whereas parasite detection revealed avian infections in these same species, challenging assumed host feeding patterns. This reflects a key methodological difference: barcoding shows recent feeding events, whereas parasite detection reveals longer-term associations, including hosts no longer present or undetectable owing to blood digestion. Together, these methods help identify potential bridge vectors and uncover new transmission pathways. In our study, we found *Ae. vexans* and *C. pictipennis* harbouring both human blood and avian parasites (*P. matutinum* and *Haemoproteus* sp.), highlighting their role in linking birds and mammals, including humans.

However, parasite detection may underestimate certain host associations owing to vector incompatibility. For example, *Culex* mosquitoes are refractory to mammalian *T. theileri* [23], so parasite-based data may suggest bird specificity, even when blood barcoding confirms mammal feeding. Conversely, rare hosts without associated parasites may go undetected by parasite screening and can be identified via barcoding. In such cases, the absence of parasite DNA reflects vector–parasite incompatibility rather than a lack of feeding.

Both methods have limitations. Mixed or ambiguous results, such as multiple host sequences or undetermined parasite lineages, were occasionally observed. PCR may underestimate co-infections owing to preferential amplification of dominant templates [101, 102]. In our study, 13 haemosporidian sequences (9.5%) could not be assigned to known lineages owing to low sequence quality. Additionally, host barcoding primers, not optimised for detecting multiple meals, may have underestimated feeding complexity. While the primers used in this study (12S3F and 12S5R) are suitable for identifying the primary host species, we acknowledge their limited ability to resolve mixed blood meals containing multiple host sources. This constraint may have led to an underestimation of complex feeding behaviour in our samples. To gain deeper insights into host feeding patterns, future studies could explore alternative methods capable of detecting multiple hosts within a single blood meal, such as multiplex PCR or advanced sequencing technologies (e.g. next-generation sequencing, NGS). NGS-based methods could help overcome current limitations by improving resolution for both host and parasite identification [103, 104]. While parasite detection often lacks host specificity, it complements barcoding by revealing broader host–parasite associations, detecting parasite infections over a longer time window than blood remains, and occasionally revealing unexpected parasite–vector associations. Together, these complementary methods provide a more comprehensive picture of host feeding patterns and parasite transmission dynamics than either approach alone.

### Additional findings and technical observations

Although specific primers for *Leucocytozoon*, a parasite transmitted by blackflies, were used, no infections were detected despite its known presence in local avian hosts ([105]; Svobodová, unpublished). Several factors may explain this absence: *Leucocytozoon* typically occurs at low parasitaemia, does not replicate in non-vector insects, and is unable to persist in the gut after ingestion, unlike some haemosporidians or trypanosomes [15].

## Conclusions

This study significantly expands the knowledge of haemosporidian parasite diversity in Czechia, identifying 15 previously unreported lineages of *Plasmodium* spp. and four *Haemoproteus* spp. lineages. Additionally, a new lineage of *P. matutinum* was characterised from *Ms. richiardii*, along with three new lineages of *P. relictum* from *Cx. pipiens*.

Two methodological approaches were used to determine the feeding patterns of mosquitoes and biting midges and to compare their effectiveness and consistency. Parasite detection provides the advantage of prolonged detection in insects, as parasites can persist or develop abortive infections even in non-competent vectors. A limitation, however, is that this method cannot reveal feedings on hosts that do not harbour the targeted parasites, e.g. humans in our case. In this regard, blood meal barcoding provides more comprehensive information about host feeding patterns. However, if only one method had been applied, important information would have been missed, as some mosquitoes or biting midges could be mistakenly considered exclusively mammalophilic or ornithophilic. Although blood meal analysis remains the primary methodology for studying insect feeding patterns, integrating parasite detection complements this approach and enhances insights into behaviours of bloodsucking insects. Notably, vector competence, which determines parasite survival and development within the vector, further emphasised the value of combining both methods for a more complete understanding of host–vector–parasite interactions. In addition, this combined approach can reveal potential bridging vectors, such as in our case detecting human blood with avian parasites.

Detecting various spectrums of parasites can provide a more comprehensive understanding. Overall, our findings demonstrate that combining blood meal barcoding with parasite detection offers a more robust and nuanced framework for understanding the feeding ecology of blood-sucking insects and the transmission dynamics of vector-borne pathogens.

## Data Availability

Data supporting the main conclusions of this study are included in the manuscript.
