# Correction: Combining blood meal analysis and parasite detection yields a more comprehensive understanding of insect host feeding patterns

**DOI:** 10.1186/s13071-025-07005-5

**Published:** 2025-08-28

**Authors:** Anna Kapustová, Magdaléna Kulich Fialová, Milena Svobodová, Jana Brzoňová

**Affiliations:** Charles University, Prague, Czechia

**Correction: Parasites & Vectors (2025) 18:288** 10.1186/s13071-025-06931-8

Following publication of the original article [1], it came to the author’s attention that the designations of novel parasite lineages (which are reported in the Results section) were detailed incorrectly in Table 5. As can be seen in the Results section (in subsection Prevalence of parasites in the insects), the correct lineage designations are CXPIP40 (accession no. PV085841), CXPIP41 (PV085842), and CXPIP42 (PV085843). The table has been corrected accordingly.

## References

[1] Kapustová A, Kulich Fialová M, Svobodová M, Brzoňová J. Combining blood meal analysis and parasite detection yields a more comprehensive understanding of insect host feeding patterns. Parasit Vectors. 2025;18:288. 10.1186/s13071-025-06931-8. 40685354 10.1186/s13071-025-06931-8 PMC12276692

